# Benchmarks for taxonomic classification of jingmenviruses and closely related viruses using newly identified genomic sequences

**DOI:** 10.1099/jgv.0.002254

**Published:** 2026-05-08

**Authors:** Agathe M. G. Colmant, Rhys H. Parry, Remi Charrel, Bruno Coutard

**Affiliations:** 1 Unite des Virus Emergents, Universita di Corsica, Marseille, France; 2 School of Chemistry and Molecular Biosciences, The University of Queensland, Brisbane, QLD, Australia

**Keywords:** arbovirus, classification, evolution, flavi-like virus, jingmenvirus, taxonomy

## Abstract

Jingmenviruses are a group of viruses related to orthoflaviviruses characterized by a segmented genome and multipartite organization that have been detected worldwide in a wide range of hosts. With the growing number of new jingmenvirus sequences identified in metagenomics data, it can be difficult to assess whether a new sequence is associated with a new virus species or with a strain of an existing species. The ICTV is about to ratify the reclassification of the *Flaviviridae* family, recognizing segmented viruses previously designated jingmenviruses as part of that family and proposing two genera to classify them: *Jingmenvirus* and *Guaicovirus*. These proposals do not include clear criteria to classify jingmenviruses and related sequences into species or genera. In order to determine such criteria, we generated a large sequence database from published and newly assembled sequences. Indeed, we screened public raw sequencing data from studies that did not search for or report jingmenvirus or related sequences, looking for new strains of previously described viruses. We then performed multiple sequence alignments and used the inferred percentage identity values to determine demarcation criteria based on the distribution of evolutionary distances upon pairwise comparisons. We report the identification of almost 60 libraries containing jingmenvirus and related sequences, in a wide range of sample types and geographical locations. Using these data and published sequences, we have determined that to be classified as a virus species, at least four segments are required, on which eight cut-off values in percentage identity (nucleotide and amino acid) are used for demarcation. The use of these criteria would enhance consistency in jingmenvirus taxonomy and provide a standardized framework for comparative genomics studies of these viruses, as they are still under-characterized.

## Data Availability

Nucleotide sequence data reported are available in the Third Party Assembly/Annotation Section of the DDBJ/ENA/GenBank databases under the accession numbers TPA: BK082815-BK083032. The details for all sequences in the database can be found in File S1, available in the online Supplementary Material.

## Introduction

Jingmenviruses are viruses with a segmented genome that have been detected all over the world in a wide range of samples, mostly by metagenomics [1]. The two segments of the genome containing coding sequences for (i) the RdRp and methyltransferase domains, as well as (ii) the helicase and protease domains, share sequence homology with members of the *Orthoflavivirus* genus in the *Flaviviridae* family. The other segments contain coding sequences for putative structural proteins and share limited sequence homology with the rest of the virus realm, although structural homology has been found between some of the encoded proteins and orthoflavivirus structural proteins [2,3]. To date, the published jingmenvirus sequences have been unofficially separated into two main clades based on phylogenetic analyses, one clade termed tick- and vertebrate-associated jingmenviruses (type species Jīngmén tick virus, JMTV) and the other clade termed insect- and plant-associated jingmenviruses (type species Guaico Culex virus, GCXV). While this designation facilitates sequence description, it is flawed in the sense that some sequences in the tick- and vertebrate-associated clade originated from mosquitoes, cattle faeces or soil metagenomic samples, while some sequences from the insect- and plant-associated clade are derived from metagenomics samples of crayfish, scorpions, water samples or even from vertebrates [1,4,6]. These sequences were not classified as of the ICTV August 2024 release and were referred to as unclassified viruses, related to the *Orthoflavivirus* genus, *Flaviviridae* family and *Amarillovirales* order in the NCBI and ICTV taxonomy browsers.

A recent proposal to the ICTV aims to reclassify the *Flaviviridae* family, in particular with the creation of two genera, including virus species with segmented genomes: *Jingmenvirus* (type species *Jingmenvirus rhipicephali*, JMTV) and *Guaicovirus* (type species *Guaicovirus culicis*, GCXV). This proposal, based on a recent study by Simmonds *et al*., should be ratified in early 2026 [7]. Classifying segmented genomes into genera within the *Flaviviridae* will be helpful to study the evolution of these viruses, but Simmonds *et al*. did not include demarcation criteria in their proposal to help classify new sequences into species. What’s more, the variability in identified segment numbers and the existence of jingmenvirus-related endogenous viral elements (EVEs) integrated in tick genomes complicates annotation of novel sequences and therefore their classification [6,8,11]. Demarcation criteria can be determined using the distribution of percentage identity upon pairwise comparison of related nucleotide or amino acid sequences [12,13]. The alignments from which these values are inferred ideally need to include sequences of different strains of the same virus, as well as sequences of different virus species, in order to determine criteria that properly differentiate species from each other. In this study, we endeavour to put forward criteria to clarify the taxonomic classification of jingmenviruses and relatives.

## Methods

### Assembling new jingmenvirus strains from publicly available sequencing libraries

We used the online Serratus.io platform to find novel jingmenvirus and related sequences in publicly available raw sequencing data from the Sequence Read Archive (SRA) [14]. We started by searching for novel JMTV-related sequences. We used the Serratus NT Search (option GenBank) for JMTV strain SY84 segment 1 (NC_024113.1), selecting only libraries that matched with a score between 3 and 100, and with a percentage identity above 75%IDnt, hoping to uncover libraries with JMTV sequences, as well as sequences of other species in the *Jingmenvirus* genus. We obtained 92 libraries that matched these criteria.

We then broadened the scope of our search and used the Serratus RdRp Search (option Family) for *Flaviviridae*, with a score criteria between 50 and 100, and a percentage identity above 90%IDnt. We obtained 9,615 libraries sorted in subfamilies numbered by the Serratus tool. We identified jingmenvirus-related subfamilies: Flaviviridae-3 (*Jingmenvirus*) and Flaviviridae-28 (Plasmopara viticola lesion-associated Jingman-like virus 1, PVLaJlV1), and the 63 associated libraries (56 for Flaviviridae-3, 7 for Flaviviridae-28) [15,17].

Finally, we aimed at finding sequences related to the *Guaicovirus* genus. We therefore selected the Serratus RdRp Search and screened for libraries in the relevant ‘Family’, entitled Unclassified-899. With the filtering criteria used (percentage identity >90% IDnt and score >50), we identified 43 libraries.

We then selected only the libraries which were not associated with publications on jingmenviruses and relatives, eliminated duplicate records generated by our different screens and assembled jingmenvirus-related sequences from 59 libraries. We used NCBI blast with its direct access to the SRA to obtain reads mapping to reference sequences, with the Megablast algorithm optimized for highly similar sequences with maximum target sequences set to the highest setting (5,000), and assembled these reads using Geneious Prime 2024.0.7. The following reference sequences were used: JMTV KJ001579–KJ001582; ALSV MH158415–MH158418; YGTV MH688529–MH688532; XJTV1 MZ244282–MZ244285; PVLaJlV1 MN551114, MN551116, BK061346, BK061347; WHAV1 KR902721–KR902724; WHAV2 KR902725–KR902728 and Ixodes ricinus NSP1 EVE OP068155. While the viral reference sequences are described in their original publications as the complete genome, we cannot comment on the certainty of those authors that all segments have been identified.

We included partial sequences in the database when the most represented segment had been assembled with at least 25 reads.

### Database of published jingmenvirus sequences

We endeavoured to build a database for all published jingmenvirus and related sequences to be used in taxonomic analysis in addition to the newly assembled sequences. In order to do so, we compiled all sequences obtained with a search on NCBI GenBank with the following keywords: jingmenvirus, jingmen-related, jingmen-like, segmented flavivirus, segmented flavi-like, flavi-like segmented virus, flavivirus-like, flavi-like, Jingmen tick virus, Mogiana tick virus, Kindia tick virus, Guangxi tick virus, Manych virus, Sichuan tick virus, Alongshan virus, Yanggou tick virus, Xinjiang tick virus, Takachi virus, Guangdong jingmen-like virus, Hainan jingmen-like virus, Pteropus lylei jingmenvirus, Peromyscus leucopus jingmenvirus and Guaico Culex virus. We then searched NCBI PubMed with the same keyword list and compiled all published sequences related to the articles uncovered by that search. We also screened all articles citing Qin *et al*. [18], the first description of a jingmenvirus, and compiled all jingmenvirus-related sequences from these articles. Finally, we used NCBI BLASTx and searched for sequences related to all jingmenvirus species identified until then, adding to our database the sequences listed in the results that slipped through the screening process.

Our database was last updated with newly published sequences on 11 April 2025.

### Percentage identity frequency distribution histograms

Nucleotide and protein sequences were aligned using MAFFT v7.490 [19] with the L-INS-i algorithm, which is recommended for sequences with conserved domains and variable regions. The L-INS-i strategy uses iterative refinement with local pairwise alignment information and is particularly suitable for datasets with multiple distinct sequence regions.

We selected only records with complete genomes (at least four coding-complete sequences) and aligned their coding nucleotide sequences, as well as their amino acid translation for all segments, except for records of GCXV segment 5 since they have no known homologues. Given the differences in nomenclature between clades, we will thereafter designate segment 1 as the one coding for NSP1; segment 2 as the one coding for nuORF, VP1, VP1ab, VP4-1 or VP4 and VP5-6; segment 3 as the one coding for NSP2; and segment 4 as the one coding for VP2-3 or VP1-3 and VP2. We exported the percentage identity matrices for all alignments and used the FREQUENCY function of Excel to generate a table of frequencies, with percentage identity values rounded to the hundredth as bins. The frequencies were plotted as distribution histograms with GraphPad Prism 9. Frequencies that are not represented in the original matrices result in gaps in the distribution, which indicates a separation between groups of sequences, and can be used as criteria for taxonomic classification [12,13].

### Phylogenetic analysis

Best-fit evolutionary models for both nucleotide and protein alignments were determined using ModelFinder [20] as implemented in IQ-TREE 3.0.1. Model selection was performed based on the Bayesian Information Criterion, which balances model fit and complexity to avoid overfitting (Table 1). For nucleotide alignments, the selected models ranged from TIM2+R4 to TIM2+F+I+R5, indicating varying levels of rate heterogeneity across different genomic segments. For protein alignments, the selected models included LG+I+R4, LG+I+R5 and JTT+I+R4, reflecting the diverse evolutionary constraints acting on different protein-coding regions.

**Table 1.** Models selected based on the Bayesian Information Criterion for phylogenetic analyses

Segment	Nucleotide	Amino acid
1 (NSP1)	TIM2+F+I+R5	LG+I+R4
2 (VP1 or VP1ab; VP4-1; VP4)	TIM2+R4	JTT+I+R4
3 (NSP2)	TIM2+I+R5	LG+I+R4
4 (VP2-3; VP1-3)	TIM2+R5	LG+I+R5

Phylogenetic trees were constructed using IQ-TREE multicore version 3.0.1 for Windows [21] with the best-fit models as determined by ModelFinder. Branch support was assessed through 1,000 ultra-fast bootstrap replicates [22] and 1,000 SH-like approximate likelihood ratio test replicates [23]. The command used for phylogenetic analysis was iqtree3 -s [alignment_file] --alrt 1000 -B 1000. The trees were visualized, mid-point rooted and formatted using the online platform Interactive Tree of Life, version 7.2.2 [24].

## Results

### New jingmenvirus-related sequences assembled from 59 libraries

In order to build a large jingmenvirus and relative genomic sequence database, we searched for undescribed sequences of known jingmenviruses from both putative clades (proposed genera) in public sequencing data. Using Serratus, we found 59 libraries with nucleic acid related to jingmenviruses that were not linked to a publication reporting such sequences and assembled full and partial genomic sequences from these libraries. Of note, libraries SRR21218428, SRR14866274, SRR14866275, SRR14866276 and SRR14866277 contain jingmenvirus-related sequences that have been partially described in publications but that were not deposited on GenBank [11,25].

Overall, we report 15 new sequences closely related to JMTV, 9 to Ālóngshān virus (ALSV), 1 to Xinjiang tick virus 1 (XJTV1), 1 to Yánggōu tick virus (YGTV), 5 to PVLaJlV1, 16 to Wǔhàn aphid virus 1 (WHAV1) and 5 to Wǔhàn aphid virus 2 (WHAV2) strains. We also identified ten libraries containing a sequence closely related to the known *Ixodes ricinus* jingmenvirus-like segment 1 EVE from *Ixodes ricinus*, and one library with a related putative jingmenvirus-like segment 1 EVE from *Ixodes pacificus*. The corresponding 59 libraries are detailed in Table 2, alongside two tick genomic sequence libraries from which we identified novel putative EVEs. We did not identify instances of more than one jingmenvirus in a single library, although certain libraries had reads mapping to both ALSV and the segment 1 EVE.

**Table 2.** Public libraries containing the genomic sequences of new strains of known jingmenviruses or jingmenvirus-derived endogenous viral elements, with metadata extracted from these public records

Library	Bioproject	Location	Date	Sample origin	Closest relative	GenBank accessions (pending)
ERR2844017, ERR2844018	PRJEB29020	South Korea	2018	Mice	JMTV	BK082853-BK082860
SRR10257635	PRJNA576495	Georgia	2012	*P. kandelakii*	JMTV	BK082861-BK082864
SRR10680474	PRJNA557323	Hong Kong	UNK	*Homo sapiens*	JMTV	BK082865- BK082868
SRR10740626	PRJNA596777	Brazil	2015	Tick	JMTV	BK082869- BK082872
SRR1745805	PRJNA271540	China	2012	Ticks	JMTV	BK082901- BK082904
SRR6912096	PRJNA446065	USA	2015	*O. turicata*	JMTV	BK082905- BK082908
SRR7786638	PRJNA489181	Türkiye	2014	Tick	JMTV	BK082909- BK082912
SRR17422335	PRJNA793118	China	2020	*R. sanguineus*	JMTV	BK082897- BK082900
SRR14127279	PRJNA718993	China	UNK	*R. microplus*	JMTV	BK082873- BK082876
SRR14127281	PRJNA718993	China	UNK	*Hae. longicornis*	JMTV	BK082877- BK082880
SRR14866274* SRR14866275* SRR14866276* SRR14866277*	PRJNA739381	Colombia	2018–2019	*R. microplus*	JMTV	BK082881- BK082896
SRR592675	PRJNA177622	Czechia	UNK	Tick	ALSV	BK082831- BK082834
SRR10569261 SRR10569265	PRJNA592700	na	na	*S. aureus* (ATCC 25923)	ALSV	BK082815- BK082822
SRR12604282	PRJNA662080	France	2016	*I. ricinus*	ALSV	BK082823- BK082826
SRR641305, SRR641306, SRR641327, SRR641328	PRJNA183509	Czechia	UNK	Tick	ALSV I. ricinus EVE	BK082835- BK082848, BK083040, BK083041
SRR12604287	PRJNA662080	Switzerland	2017	*I. ricinus*	ALSV I. ricinus EVE	BK082827- BK082830
SRR3170015, SRR3170023	PRJNA311553	Czechia	UNK	Tick	I. ricinus EVE	BK083038, BK083039
ERR4666641 ERR4666644	PRJEB40724	France	2017	*I. ricinus*	I. ricinus EVE	BK083035, BK083036
SRR10740640	PRJNA589581	Czechia	2019	*I. ricinus*	I. ricinus EVE	BK083037
SRR21218428*	PRJNA870442	USA	2018	*I. pacificus*	I. pacificus EVE	BK083033
CBDIYE010047735	PRJEB89832	China	UNK	*I. persulcatus*	I. persulcatus EVE	BK083034
JABSTR010000005.1	PRJNA633311	China	2017	*Hae. longicornis*	Hae. longicornis EVE2	BK0830
SRR6371193	PRJNA422202	China	UNK	Rodent	XJTV1	BK083025- BK083028
SRR6659068	PRJNA432323	China	UNK	Tick	YGTV	BK083029- BK083032
SRR17207994, SRR17207995, SRR17207997, SRR17208000, SRR17208001	PRJNA787359	China	UNK	*Vitis vinifera*	PVLaJlV1	BK082913- BK082932
SRR11563875 SRR11563879	PRJNA625990	India	na	*Triticum aestivum*	WHAV1	BK082933- BK082940
SRR13259171	PRJNA684037	South Korea	2020	*Avena sativa*	WHAV1	BK082941- BK082944
SRR13638192	PRJNA699715	China	na	*Aphis gossypii*	WHAV1	BK082945- BK082948
SRR19974249	PRJNA855531	China	2019	*Phlebotomus chinensis*	WHAV1	BK082953- BK082956
SRR21857638	PRJNA889322	na	na	*T. aestivum*	WHAV1	BK082957- BK082960
SRR22075881 SRR22075882 SRR22075883	PRJNA894808	South Korea	na	*Pyrus pyrifolia+Venturia nashicola*	WHAV1	BK082961- BK082972
SRR5831395, SRR5831423	PRJNA394582	Kenya	2014	*Aphis fabae*	WHAV1	BK082973- BK082980
SRR7724999	PRJNA486859	China	na	*Malus neidzwetzkyana*	WHAV1	BK082981- BK082984
SRR7813612, SRR7813619, SRR7813620, SRR7813651	PRJNA290156	na	na	*Lysiphlebus fabarum+Aphis fabae*	WHAV1	BK082985- BK083000
SRR14663377	PRJNA732832	na	na	*Zanthoxylum armatum (leaf*)	WHAV1	BK082949- BK082952
ERR7672962	PRJEB49313	UK	2017	Agricultural soil	WHAV2	BK083001- BK083004
SRR19573187, SRR19573188, SRR19573189	PRJNA826712	na	na	*Acyrthosiphon pisum*	WHAV2	BK083005- BK083016
SRR6239954	PRJNA415643	na	2016	*Buchnera aphidicola*	WHAV2	BK083017- BK083020

*Jingmenvirus sequence partially described in a publication but sequence not deposited to GenBank.

Hae, *Haemaphysalis*; I, *Ixodes*; na, not applicable; O, *Ornithodoros*; P, *Phlebotomus*; R, *Rhipicephalus*; S, *Staphylococcus*; UNK, unknown.

### Building jingmenvirus sequence databases

Using the methods described below, we collated a database of 1948 published jingmenvirus-related sequence records across all segments and added the viral sequences we assembled here for a total of 2,175 sequences (Table 3 and File S1). Six hundred and sixty-three were for the segment coding for the RdRp and methyltransferase domains (NSP1), 538 were for the segment coding for the putative glycoprotein (nuORF, VP1, VP1ab, VP4-1 or VP4 and VP5-6), 546 were for the segment coding for the helicase and protease domains (NSP2), 421 were for the segment coding for the putative membrane protein (VP2-3 or VP1-3 and VP2), and 5 were for the fifth segment detected solely in GCXV (VP7, thereafter excluded from the analyses). Out of these records, 327, 328, 315 and 306 were coding-complete sequences of the 4 segments described above, respectively, out of which 4×263 represented putative complete genomes, which we define here as at least 4 segments and coding-complete sequences for each segment.

**Table 3.** Number of jingmenvirus-related sequences in the database

Protein encoded (segment no.)	Nucleotide (complete and incomplete)	Coding-complete sequences	Complete genome
NSP1 (1*,†,‡)	602+**61**=663	279+**48**=327	223+**40**=263
nuORF+VP1 or VP1ab (2)* VP4-1 (2)† VP4+VP5-6 (4)‡	485+**56**=538	286+**42**=328	
NSP2 (2‡ or 3*,†)	493+**56**=546	272+**43**=315	
VP2-3 (4*,†) VP1−3+VP2 (3)‡	368+**54**=421	262+**44**=306	

Underlined: published; in bold: assembled in this study. Nomenclature for *tick- and vertebrate-associated, †insect-associated and ‡mosquito-derived jingmenviruses.

Given the differences in nomenclature between clades, we will thereafter designate segment 1 as the one coding for NSP1; segment 2 as the one coding for nuORF, VP1, VP1ab, VP4-1 or VP4 and VP5-6; segment 3 as the one coding for NSP2; and segment 4 as the one coding for VP2-3 or VP1-3 and VP2.

### Proposing taxonomic classification criteria derived from %ID distribution histograms

**Fig. 1.**
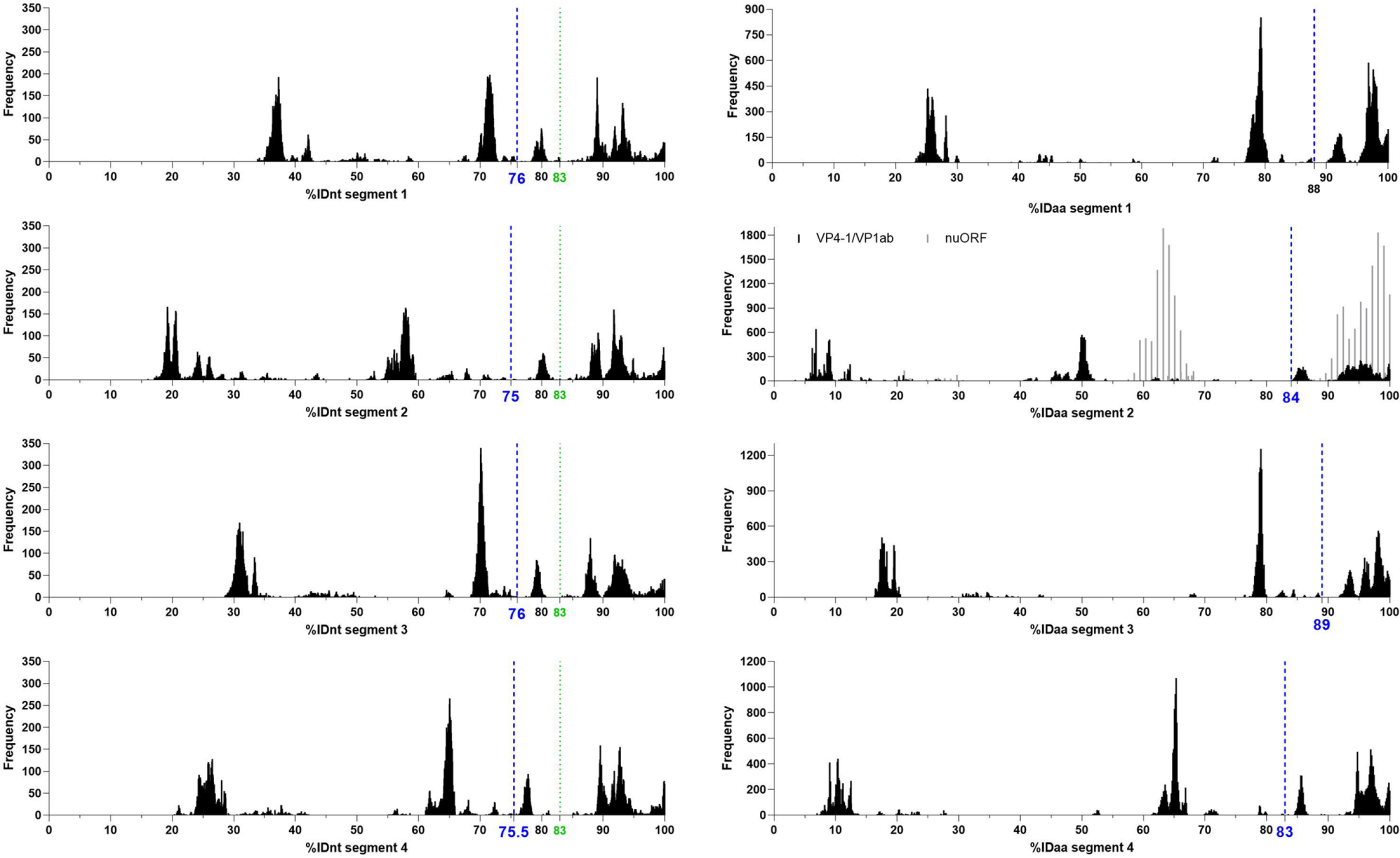
Frequency distribution histograms of percentage identities between jingmenvirus sequences for segments 1–4, in nucleotide and amino acid. Blue dashed vertical lines represent the determined demarcation criteria for species, and green dotted vertical lines represent the determined rule for genotypes within species. *n*=263 for all alignments except nuORF, for which *n*=211.

**Fig. 2.**
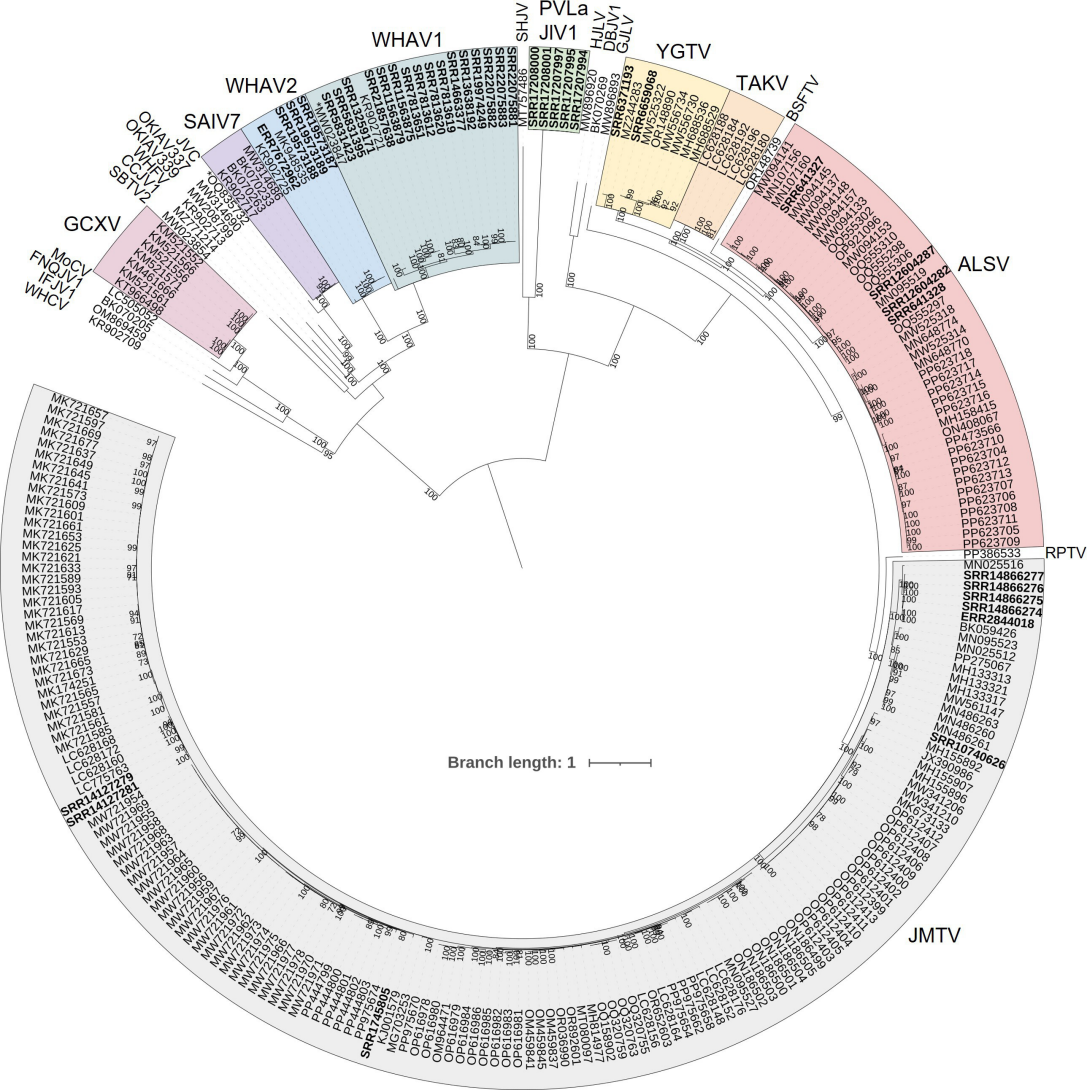
Phylogenetic analysis of segment 1 complete coding sequences (whole genomes only, i.e. including at least four segments; *n*=263). The sequences were aligned with MAFFT in Geneious Prime 2024.0.7, and the phylogeny was built with IQ-TREE3, using the TIM2+F+I+R5 model, and mid-point rooted using Interactive Tree of Life. Bootstrap support values above 70 were included as branch labels. The scale bar represents substitutions per site. The sequences identified by our criteria as part of the same species are highlighted in differently coloured ranges. Sequences that are not in a coloured range represent the only complete genome of their species. The two sequences which we could not conclusively classify using our criteria are preceded by an asterisk (*). The sequences assembled in this study are highlighted in bold. JMTV, Jīngmén tick virus; RPTV, Rio Preto virus; ALSV, Ālóngshān virus; BSFTV, Báishān forest tick virus; TAKV, Takashi virus; YGTV, Yánggōu tick virus; GJLV, Guǎngdōng jingmen-like virus; DBJV1, Dysdera bandamae jingmenvirus 1; HJLV, Hǎinán jingmen-like virus; PVLaJlV1, Plasmopara viticola lesion-associated Jingman-like virus 1; SHJV, Solling Histiostoma jingmenvirus; WHAV1, Wǔhàn aphid virus 1; WHAV2, Wǔhàn aphid virus 2; SAIV7, Shuāngào insect virus 7; JVC, Jingmenvirus Cameroon (unclassified); OKIAV337, Trichopteran jingmen-related virus; OKIAV339, Neuropteran jingmen-related virus; WHFV, Wǔhàn flea virus; CCJV1, Culicoides circumscriptus jingmenvirus 1; SBTV2, Soybean thrips virus 2; GCXV, Guaico Culex virus; MoCV, Mole Culex virus; FNQJV1, Far North Queensland jingmenvirus 1; IFJV1, Inopus flavus jingmenvirus 1; WHCV, Wǔhàn cricket virus.

The sequences from the 263 complete genomes were used to perform multiple sequence alignments for each segment in nucleotide and amino acid, from which percentage identities (%ID) were inferred. We then derived frequency distribution histograms from the %ID matrices generated. Gaps in this distribution separate groups of viruses that share similar %ID and can denote demarcation criteria to be used to differentiate between virus species (Fig. 1). We identified several possible values that could be used as criteria within the distributions obtained. Guided by the phylogenetic trees (Fig. 2 and File S2) derived from the same alignments and by metadata (assigned viral species name, host and location) informing on the biology linked to the sequences, we determined which values were the most relevant to use as criteria to classify sequences into virus species, for all segments, in nucleotide %ID (%IDnt) and %ID amino acid (%IDaa). We aimed at selecting the most parsimonious threshold values that would result in all four segments of a genome being classified the same using the eight %IDnt and %IDaa criteria.

In nucleotide, we recommend that sequences sharing <76%IDnt in segment 1, <75%IDnt in segment 2, <76%IDnt in segment 3 and <75.5%IDnt in segment 4 with their closest relative should be considered a novel species (see Table 4). Taking the segment 1 nucleotide histogram as reference, the other main peaks correspond, in broad strokes, to %ID between different species within the *Jingmenvirus* genus (~60–76%IDnt), between species within the *Guaicovirus* genus (~45–60%IDnt) and between species of different genera (<45%IDnt).

**Table 4.** Species demarcation criteria in percentage identity (nucleotide and amino acid)

	Segment 1	Segment 2	Segment 3	Segment 4
**Species (nt)**	76%	75%	76%	75.5%
**Species (aa)**	88%	84%	89%	83%

In amino acid, we recommend that sequences sharing <88%IDaa in segment 1, <84%IDaa in segment 2, <89%IDaa in segment 3 and <89%IDaa in segment 4 with their closest relative should be considered a novel species (see Table 4).

We recommend using all species criteria when possible, given the segmented and multipartite nature of jingmenviruses, and the associated probability of segment reassortment and/or recombination.

### Applying taxonomic species criteria to our database and investigating classification discrepancies

As we recommend the use of the eight %ID species criteria (see Table 4), we applied them all to our complete and partial sequences database and found discrepancies between the species our analysis would assign and some sequences’ published names. We therefore investigated these discrepancies to determine whether our criteria were inaccurate or if they would support re-classification of some published sequences.

#### Grouping sequences published with different names into one species

We found that many sequences associated with various virus names shared %ID way above our recommended thresholds both in nucleotide and amino acid (Table 4) and shared common hosts and/or locations, which would be consistent with these sequences belonging to a single species. In that case, sequences labelled JMTV, Mogiana tick virus, Amblyomma virus, *Rhipicephalus*-associated flavi-like virus, Kindia tick virus, Sichuan tick virus, Guangxi tick virus and Manych virus would all belong to the same species (*J. rhipicephali*). Following the same rationale, sequences named ALSV and Harz mountain virus would be included in the same species; sequences labelled Havel Jingmen-like virus and Teltow Canal Jingmen-like virus would be grouped into one species; sequences labelled Wǔhàn flea virus (WHFV) and Siphonapteran jingmen-related virus isolate OKIAV340 (published and assembled here MW208795, MW208800; BK083021- BK083024) would be grouped into one species; sequences labelled XJTV1 would be grouped into the same species as YGTV sequences, as they fit all nucleotide and amino acid criteria, with only one exception (VP1 81.8%IDaa, thus <84.0%).

#### Re-classifying to another species

We found sequences sharing >99%IDnt and aa with species that did not correspond to the names they were assigned, so according to our criteria, these sequences would be reclassified. Indeed, MN095519–MN095522 and MH678646–MH678648 (labelled JMTV) share >99%ID nt and aa for all segments with strains of ALSV; MG880118–MG880119 (labelled JMTV) share >99%ID with strains of YGTV; OP376740 (labelled YGTV) shares >99%ID with strains of ALSV.

We found that MH155900 is mislabelled as JMTV segment 2 but represents a segment 3 sequence.

Then, we found sequences labelled JMTV that shared %ID below all recommended threshold with all described jingmenvirus sequences, which would be classified as new species following our criteria: we recommend designating MN095531–MN095534 as Pteropus lylei jingmenvirus, and MT747997–MT748000 and MT757486 as two strains of Solling histiostoma jingmenvirus. Similarly, OP863303 (labelled JMTV segment 1) shares less than 76%IDnt with JMTV and is more closely related to ALSV, but due to there being only one segment available, we will abstain from classifying it (see below).

#### Re-classifying EVEs mislabelled as virus

Moreover, we have found sequences labelled as virus that belong to known and previously unidentified EVEs. MT822179, MT822180, OQ162438 (labelled JMTV) and ON933885–ON933950 (labelled Jingmenvirus *sp* isolate) share >95%IDnt with the previously described *I. ricinus* jingmenvirus-like segment 1 EVE. We found that MZ676705 (labelled ALSV Liaoning; 2.5 kb) and OR114850–OR114911 (labelled Cheeloo jingmen-like virus; up to 2.7 kb) share >95%IDnt with each other. Since these were the only jingmenvirus-like segment sequences assembled from these samples, we investigated whether they could be integrated in their host genome, based on the existence of the *I. ricinus* jingmenvirus-like segment 1 EVE. In the absence of samples on which to perform molecular analyses, we searched for these sequences using blast on the tick’s whole-genome sequences (WGS NCBI database) and found that these sequences indeed corresponded to a jingmenvirus-like segment 2 EVE integration in the *Hae. longicornis* genome. We performed a similar analysis on other single-segment sequences for which the host genome had been sequenced and was available and found that OQ716542 (labelled ALSV; 373 bp) is part of another partial segment 2-derived EVE in the *Hae. longicornis* genome, for which we assembled a 1.7 kb contig (BK083042), and which shares 79.0%IDnt with the ‘Liaoning/Cheeloo’ EVE. We found that OP863304 (labelled JMTV; 386 bp), OP244356, OP244397–402, OP244412–14 and PQ043852 (labelled ALSV; 219–332 bp) are part of a jingmenvirus-like segment 4-derived integration in the *I. persulcatus* genome for which we were able to assemble a 2.6 kb contig (BK083034). It is important to note that jingmenvirus-derived EVEs can share %ID above our recommended thresholds with jingmenvirus species. For example, the *I. ricinus* jingmenvirus-like segment 1 EVE and *I. pacificus* jingmenvirus-like segment 1 EVE (BK083034) both share >76%IDnt with ALSV segment 1. However, considering these sequences are not viral RNA but a DNA form integrated in the tick genome, they should be classified as non-viral sequences and therefore not associated with any jingmenvirus species at this stage. The biology of the analysed sequences should be taken into account alongside the %ID criteria when classifying sequences, and the description of novel jingmenvirus species should include the sequences of at least four segments to avoid these types of errors.

#### A minority of unclassifiable sequences

For a few sequences, the discrepancies between the published species names and our criteria-derived results were not as straightforward to resolve. Indeed, the eight recommended %ID criteria did not allow the clear classification of three sets of published sequences. First, for MW023847–MW023850, labelled WHAV1, the %IDnt for the four segments is right at the species threshold either just below or just above it, while the four %IDaa criteria would classify the sequences as belonging to a new species. Second, for OQ835732–OQ835735 and BK070268, labelled Jingmenvirus Cameroon, some criteria would have it classified as belonging to the same species (segment 1, 2-1, 2-2 and 3 nt; segment 2-2 aa) as its closest relative, Shuāngào insect virus 7, while the other criteria would have it classified as another species (segment 4 nt; segment 1, 2-1, 3 and 4 aa). Third, the sequence OP863303 (labelled JMTV segment 1) would belong to a novel jingmenvirus species based on the %IDnt criterion, but its NSP1 ORF shares 100%IDaa with an ALSV strain (QHW06950). We intended to find other segments in the associated libraries to help with classification, but we did not find any reads mapping to this sequence, so it is unclear how the authors assembled it in the first place [26]. Considering there was a single segment published, we searched for but found no evidence that this sequence could be integrated in the *I. persulcatus* genome with blast wgs.

The recommended criteria allowed to identify these sequences as outliers, but with the current lack of published data on the biology of these viruses, it is not clear yet how they should be classified or why these sequences are outliers. Characterizing the viruses in question would allow us to definitively conclude on the matter.

Considering that the ten unclassifiable sequences described in the previous paragraph represent a small minority within the large 2180 sequence database, we are satisfied with the performance of the eight recommended criteria, with the addition of the recommendation to have at least four segment sequences when describing a new species of flavivirus with a segmented genome.

### Species criteria with >99.6% accuracy performance

We endeavoured to confirm the performance of the recommended eight species criteria by measuring their accuracy on the coding-complete sequence database. In order to do so, we compared the %IDnt and %IDaa of each sequence with every other sequence in the database (not only their closest relative) and counted how many did not fit the criteria properly: for example, in the segment 1%IDnt matrix, how many %ID were below 76% in members of the same assigned viral species and how many were above between members of different species. We then calculated the percentage accuracy of each criterion by comparing that number to the total number of entries in each %ID matrix. We classified the sequences according to our criteria and chose a reference for the outlier sequences (MW023847–MW323850 sequences considered as WHAV1; OQ835732–OQ835735 and BK070268 as their own species). With these assignments, the accuracy of the method is >99.6% for all criteria (Table 5).

**Table 5.** Classification criteria accuracy. Number of %ID value outside of the recommended criteria in each %ID matrix, based on the CCDS database, and corresponding percentage accuracy for each criterion

	Segment 1	Segment 2	Segment 3	Segment 4
Number of sequences in database	327	328	315	306
Nucleotide criteria accuracy	89/53,301 99.83%	7/53,628 99.99%	30/49,455 99.94%	21/46,971 99.96%
Amino acid criteria accuracy	166/53,301 99.69%	188/53,628 99.65%	181/49,455 99.63%	28/46,971 99.94%

### Identifying several species with two genotypes/lineages

Considering that we and others have described numerous and sometimes divergent strains for a few jingmenvirus and related species, we also put forward a rule for genotype/lineage identification with a threshold at 83%IDnt for all segments, separating certain species into sub-species. While this rule is out of the scope of taxonomical considerations, it will benefit this research community by clarifying the spatial and temporal distribution, as well as host association of divergent strains of the same viral species. We opted to number putative genotypes (I and II) based on the chronology of their publication: earlier sequences being assigned to genotype I.

Following this rule, we found that JMTV sequences could be grouped into two genotypes. JMTV genotype I would include complete and partial sequences from Asia (China, Japan, Laos and Hong Kong), Africa (Kenya, Uganda, Guinea and Cameroon), Europe (Georgia, Türkiye and Russia) and the Americas (Brazil and USA). The JMTV genotype I sequences would originate from a wide range of hosts: arthropods such as ticks (genera *Amblyomma*, *Dermacentor*, *Haemaphysalis*, *Hyalomma*, *Ixodes* and *Rhipicephalus*, as well as the soft tick *Ornithodoros turicata*), mosquitoes (*Armigeres* sp.) sand flies (*Phlebotomus kandelakii*) and vertebrates such as rodents (genera *Apodemus*, *Cricetulus*, *Meriones*, *Microtus*, *Mus*, *Rattus* and *Rhombomys*), bats (genera *Eptesicus*, *Miniopterus*, *Myotis*, *Nyctalus*, *Pipistrellus* and *Rhinolophus*), cattle (*Bubalus bubalis* and *Bos taurus*), primates (*Piliocolobus rufomitratus* and humans) and reptiles (*Stigmochelys pardalis*). JMTV genotype II would include complete and partial sequences from Europe (Kosovo, Romania, Türkiye, Italy, France and Russia), the Americas (Trinidad and Tobago, Guadeloupe/Martinique, Colombia and Mexico) and Asia (South Korea; although it is not clear whether the sequence originated from laboratory or environmental sample). The sequences grouped in this JMTV genotype would originate from different host types: tick species such as *R. microplus*, *R. bursa*, *R. sanguineus*, *R. turanicus*, *H. marginatum*, *I. simplex* and *Am. variegatum*; other arthropods such as *Ae. albopictus* from a laboratory colony and *Ochlerotatus caspius*; human patients; and mouse intestines of unknown species and origin (laboratory or environmental). Strikingly, the segments of a given JMTV strain all fall in the same genotype. Moreover, we found only one study with detections of both JMTV genotypes in tick-derived samples from the same country (Türkiye) with 2 out of 13 strains partially sequenced over segment 3 belonging to JMTV genotype II and the rest belonging to JMTV genotype I [27]. Additionally, two separate studies have found partial segment 2 sequence of both JMTV genotypes in Russia (MN218697 and MN218698 JMTV genotype I, PQ310748 JMTV genotype II). These are the only two countries to date with both JMTV genotypes recorded.

While seven out of eight %ID criteria would group sequences formerly identified as YGTV and XJTV1 into a single species, they share %IDnt that would classify them as two putative genotypes, with particularly divergent VP1 sequences, which could have evolved under different environmental pressures. The sequences formerly identified as YGTV would form genotype I, and the sequences formerly identified as XJTV1 would form genotype II. YGTV genotype I would have been detected in *D. nutalli*, *D. marginatus*, *D. reticulatus*, *D. silvarum* and *I. persulcatus* ticks collected from Russia and China. YGTV genotype II would have been detected in rodents and ticks collected from China.

Similarly, while our eight %ID criteria would group sequences formerly identified as WHFV and OKIAV340 into a single species, their genetic distance would classify them as two distinct genotypes. The sequences formerly identified as WHFV would form genotype I, and the sequences formerly identified as OKIAV340 would form genotype II. WHFV genotype I would have been detected in *Ctenocephalides felis* from China, and WHFV genotype II would have been detected in the same host collected from the USA.

While all GCXV isolates are clearly part of the same species, one strain (KM521571–KM521574) has divergent segments 2, 3 and 4 that would be classified as a different genotype, which would suggest that this isolate should be characterized to determine whether these nucleotide differences in three out of five segments result in phenotypical alterations.

### Phylogenetic analysis is in accordance with %ID criteria

We built phylogenetic trees for the sequences from complete genomes using the coding-complete sequence alignments described above (Fig. 2 and File S2) to visualize and validate the relevance of the recommended demarcation criteria. It is clear in this representation of evolutionary distance that the sequences classified as the same species are closely related and that the sequences classified as separate species are evolutionarily distant. We recommend classifying 24 jingmenvirus-related species corresponding to the genomes with at least four complete segments which are sufficiently different from each other and from their closest relatives, according to our recommended criteria (Table 6 and File S1). Among these 24 species, 9 species include several complete genomes (see coloured ranges in Fig. 2 and File S2). We did not include criteria for classification into genera as that analysis would need to include all members of the *Flaviviridae* family and is out of the scope of this study.

**Table 6.** List of jingmenvirus species based on our recommended taxonomic criteria

Species	Virus name	Reference genome
*Species #1*	Culicoides circumscriptus jingmenvirus 1	MZ771211.1–MZ771214.1
*Species #2*	Far North Queensland jingmenvirus 1	BK070205.1–BK070210.1
*‘_Guaicovirus culicis_’*	Guaico Culex virus	KM461666.1–KM461669.1
*Species #3*	Inopus flavus jingmenvirus 1	OM869459.1–OM869462.1
*Species #4*	Mole Culex virus	LC505052.1–LC505055.1
*Species #5*	Neuropteran jingmen-related virus OKIAV339	MW208799.1, MW208801.1, MW208805.1, MW208806.1
*Species #6*	Shuāngào insect virus 7	KR902717.1–KR902720.1, BK070217.1
*Species #7*	Soybean thrips virus 2	MW023854.1–MW023857.1
*Species #8*	Trichopteran jingmen-related virus OKIAV337	MW314690.1–MW314693.1
*Species #9*	Wǔhàn aphid virus 1	KR902721.1–KR902724.1
*Species #10*	Wǔhàn aphid virus 2	KR902725.1–KR902728.1
*Species #11*	Wǔhàn cricket virus	KR902709.1–KR902712.1
*Species #12*	Wǔhàn flea virus	KR902713.1–KR902716.1
*Species #13*	Ālóngshān virus	MH158415.1–MH158418.1
*Species #14*	Báishān forest tick virus	OR148739.1–OR148742.1
*Species #15*	Dysdera bandamae jingmenvirus 1	BK070269.1–BK070273.1
*Species #16*	Guǎngdōng Jingmen-like virus	MW896893.1–MW896896.1
*Species #17*	Hǎinán jingmen-like virus	MW896920.1–MW896923.1
*‘_Jingmenvirus rhipicephali_’*	Jīngmén tick virus	KJ001579.1–KJ001582.1
*Species #18*	Rio Preto tick virus	PP386533.1–PP386536.1
*Species #19*	Takachi virus	LC628180.1–LC628183.1
*Species #20*	Yánggōu tick virus	MH688529.1–MH688532.1
*Species #21*	Plasmopara viticola lesion-associated Jingman-like virus 1	BK082917- BK082920
*Species #22*	Solling Histiostoma jingmenvirus	MT747997.1–MT748000.1

The full genome sequences we were not able to classify with our criteria (Jingmenvirus Cameroon and WHAV1-like) were included in the phylogenetic analyses (preceded by an asterisk on the figure) and were indeed in intermediary positions on the tree, in the same clade as their closest relative but a bit more distant than the other members of the species, and this distance varied between segments and between nucleotide and amino acid analyses (Fig. 2 and File S2).

The whole-genome sequences included in these analyses for which we suggest grouping in two genotypes (JMTV and YGTV) have two clear clusters in their clade, corresponding to our genotype proposals.

## Discussion

While this manuscript was in its final stages of submission, a proposition to reorganize the taxonomy of the *Flaviviridae* was published by the corresponding ICTV Study Group [7]. In that study, the authors propose to organize the current *Flaviviridae* in 3 families (*Flaviviridae*, *Pestiviridae* and *Hepaciviridae*), which would include 14 genera. Two of the new genera would include segmented flavi-like virus sequences, the proposed *Jingmenvirus* genus with *J. rhipicephali* (JMTV) as a single recognized species and the *Guaicovirus* genus with *G. culicis* (GCXV) as a single recognized species, both part of the revised *Flaviviridae* family. This shows that there is indeed a great need to clarify the organization of flavi-like viruses and their taxonomy, and the criteria we recommend complement these proposals perfectly.

First, we report the detection of new sequences from several jingmenvirus-related strains, extending the known spatial and host range for these virus species. These new sequences were assembled from publicly available raw sequencing data published by others. The use of these results demonstrates the importance of open access data publication, with accurate associated metadata. By combining these newly assembled sequences with the existing ones, we collated a database that reached critical mass for addressing the taxonomic organization rules of jingmenviruses and relatives. Indeed, we have determined eight %ID species criteria (Table 4), for four segments in nucleotide and amino acid which we recommend could be used to help taxonomically classify jingmenvirus sequences into species. We move that four complete segment sequences sharing less than 75–76%IDnt and/or 83–89%IDaa (depending on the segment, see Table 4) with their closest relative could be considered as a novel jingmenvirus species. The species cut-off values determined in this study are lower than those historically applied to members of the *Orthoflavivirus* genus (84%IDnt over the whole genome) [28]. This suggests that while these groups of viruses are related, as they share genetic similarity over the segments coding for non-structural proteins, jingmenviruses have a wider diversity than orthoflaviviruses.

We are proposing %ID criteria that enabled us to (i) clearly identify 24 currently published jingmenvirus species, (ii) rationally group sequences labelled with different species names into a single species, (iii) recommend to re-classify sequences labelled with the name of another viral species than their closest relative, (iv) identify EVEs from tick genomes and (v) single out sequences with peculiar profiles which would benefit from *in vitro* characterization. The existence of criteria brings some level of order to this group of viruses and will help avoid labelling errors being perpetuated in various publications.

Beyond taxonomic classification, a rule for genotypes/lineages has enabled us to show that the widely accepted notion that jingmenvirus-related sequences group together based on their location might not be completely accurate [27,29]. Indeed, we found that both JMTV genotypes have been detected in Türkiye and Russia, from the same location and the same tick species, and that both YGTV genotypes have been detected in China [27,30]. Moreover, with JMTV sequences separated into two groups, we have been able to look for reassortments between the two genotypes and found no evidence of this phenomenon. This is not unexpected, considering that out of all studies reporting the detection of JMTV, only one has found both genotypes in the same location and tick species [27]. It therefore seems that interactions between the two JMTV genotypes, while possible, are uncommon. Similarly, the two WHFV putative genotypes were detected from the same host (*C. felis*) but from different locations (China and USA), so there is no evidence that they share an ecological niche which would facilitate reassortment. The two YGTV putative genotypes have been found in China, but it is unclear whether they share a host, as genotype II was found in samples with metadata listing ‘tick’ and ‘rodent’ hosts, and genotype I was found only in ticks, of the *Dermacentor* and *Ixodes* genera or unspecified genus. What is clear is that to date, no reassortment was observed between genotypes of any of these jingmenvirus-related species, irrespective of whether they share ecological niches or not [31].

We believe that proposing and using the eight %ID species criteria (four segments, nt and aa) is essential, in particular since jingmenviruses and relatives have a segmented genome and are thought to be multipartite. Indeed, when investigating discrepancies between published names and our analysis, we identified outlier sequences with different genetic distance profiles which could be linked to mechanisms that drove their evolution. For example, sequences closely related in amino acid and more divergent in nucleotide could be the product of constraints from different hosts shaping the nucleotide composition and codon usage, while the constraints on protein function and therefore amino acid sequence remain. Or, finding a discrepancy of genetic distance between structural vs non-structural genes could highlight evolutionary pressures acting only on selected aspects of the viral life cycle. Moreover, jingmenviruses and relatives are multisegmented and multipartite, meaning that they would encapsidate less fragments than the complete genome and that multiple particles would be required for infection [32,34]. With this type of organization, it is conceivable that one divergent segment could become part of a jingmenvirus infectious unit through co-infection or intra-host evolution [6]. Identifying such profiles is key to understanding the evolutionary mechanisms, life cycle and biology of jingmenviruses and relatives.

We have uncovered multiple instances of single-segment sequences being labelled with jingmenvirus-related species names, when in reality, they were integrated in their tick host genome. When using only the recommended %ID criteria, these sequences could indeed have passed as strains of existing species, but uncovering and taking into account their biological origin was key to classifying these sequences correctly. We therefore recommend obtaining at least four segments to consider that the new sequences indeed form a viral genome, as the currently recognized minimal number of segments for an infectious unit is four [6,32, 35]. We move that novel jingmenvirus-related sequences with less than four segments should not be labelled as virus species but rather as virus-related sequences.

Another reason to look for more than one jingmenvirus-related segment in sequencing data before labelling or classifying it would be to find out whether the sequence could be a previously undetected part of an existing genome, as we found in a previous study. Indeed, jingmenviruses can have up to at least six segments, with pairs of segments coding for homologous structural proteins [6]. In particular, in a previous study, we uncovered a previously unknown fifth segment for SAIV7 and Jingmenvirus Cameroon, homologous to their respective segment 2. The fluid genomic organization of jingmenviruses and relatives could complicate their classification, considering that in all documented cases, the two homologous segments from the same species are sufficiently genetically distant to be considered as different species, when compared. This should be taken into account, and particular attention to assembling as complete a genome as possible should be paid when describing new jingmenvirus-related sequences. A multisegmented multipartite organization facilitating segment duplication, loss or acquisition, intra- and inter-host evolution or reassortment complicates the definition of a jingmenvirus species.

The criteria we recommend and use here are based on current knowledge of jingmenvirus and relative genomic organization and biology. They are susceptible to change when further characterization and discoveries lead to a better understanding of these viruses. A great example of re-classification based on further knowledge is jiviviruses, which were originally thought to include virga-like and jingmen-like segments coding for non-structural proteins, and segments coding for putative structural proteins of unknown origin [15,36]. With the significant increase of sequencing data in the era of viromics, these considerations have since been revised, and jivivirus non-structural proteins have been shown to be more closely related to the monopartite families *Potyviridae* and *Flaviviridae* [16]. The existence of such viral genomes borrowing segments or domains from different viral families is the perfect example of why multiple criteria should be met to classify new sequences as a novel jingmenvirus-related species.

In conclusion, based on the current state of the art, we recommend only naming novel *Flaviviridae* with segmented genomic sequences as novel species when at least four segments have been sequenced, and they share <76%IDnt and <88%IDaa in segment 1, <75%IDnt and <84%IDaa in segment 2, <76%IDnt and <89%IDaa in segment 3 and <75.5%IDnt and <83%IDaa in segment 4 with their closest relative.

## Supplementary material

10.1099/jgv.0.002254 Uncited Table S1.

10.1099/jgv.0.002254 Uncited Supplementary Material 1.

## Abbreviations

ALSV: Ālóngshān virus
EVEs: endogenous viral elements
GCXV: Guaico Culex virus
%IDaa: percentage identity in amino acid
%IDnt: percentage identity in nucleotide
JMTV: Jīngmén tick virus
PVLaJlV1: Plasmopara viticola lesion-associated Jingman-like virus 1
SRA: Sequence Read Archive
WHAV1: Wǔhàn aphid virus 1
WHAV2: Wǔhàn aphid virus 2
WHFV: Wǔhàn flea virus
YGTV: Yánggōu tick virus
