# Supplementary material for: Benchmarks for taxonomic classification of jingmenviruses and closely related viruses using newly identified genomic sequences
Source: J Gen Virol. 2026 May 8;107(5):002254. doi: 10.1099/jgv.0.002254 (PMC13155725; doi:10.1099/jgv.0.002254)
Supplement: Uncited Supplementary Material 1. [file jgv-107-02254-s002.pdf]

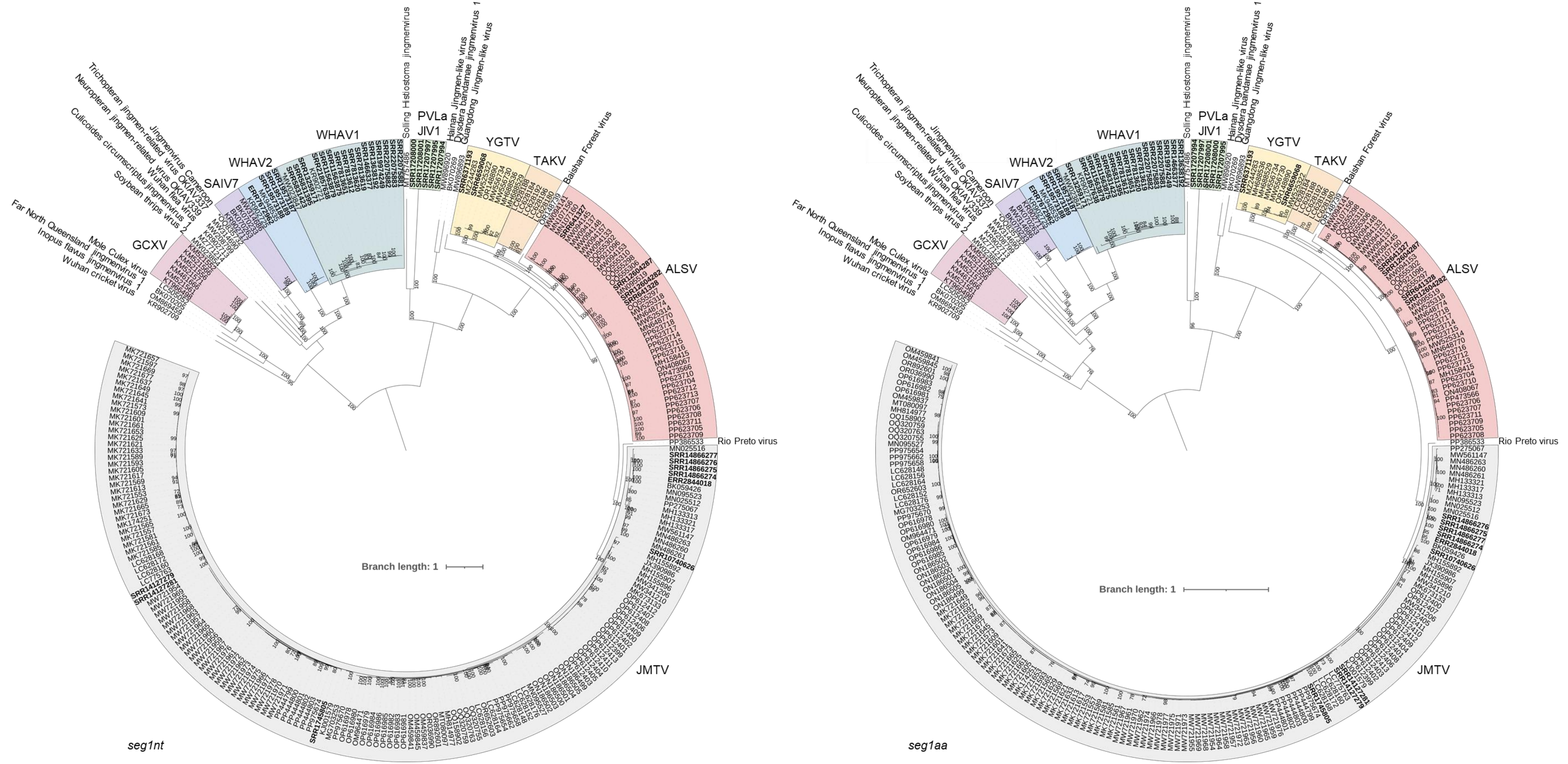

Supplementary File 2: Phylogenetic analyses of segments 1, 2 , 3 and 4 coding-complete nucleotide and amino acid sequences (whole genomes only, i.e. including at least 4 segments; n=263). The sequences were aligned with MAFFT in Geneious Prime 2024.0.7, the phylogeny was built with IQ-TREE3, using the model described in Table 5 respectively and mid-point rooted using Interactive Tree of Life. Bootstrap support values above 70 were included as branch labels. The scale bar represents substitutions per site. The sequences identified by our criteria as part of the same species are highlighted in differently coloured ranges. Sequences that are not in a coloured range represent the only complete genome of their species. The two sequences which we could not conclusively classify using our criteria are preceded by an asterisk *. The sequences assembled in this study are highlighted in bold. JMTV: Jīngmén tick virus, ALSV: Ālóngshān virus, TAKV: Takashi virus, YGTV: Yánggōu tick virus, PVLaJIV1: Plasmopara viticola lesion associated Jingman-like virus 1, WHAV1: Wǔhàn aphid virus 1, WHAV2: Wǔhàn aphid virus 2,SAIV7: Shuāngào insect virus 7, GCXV: Guaico Culex virus.

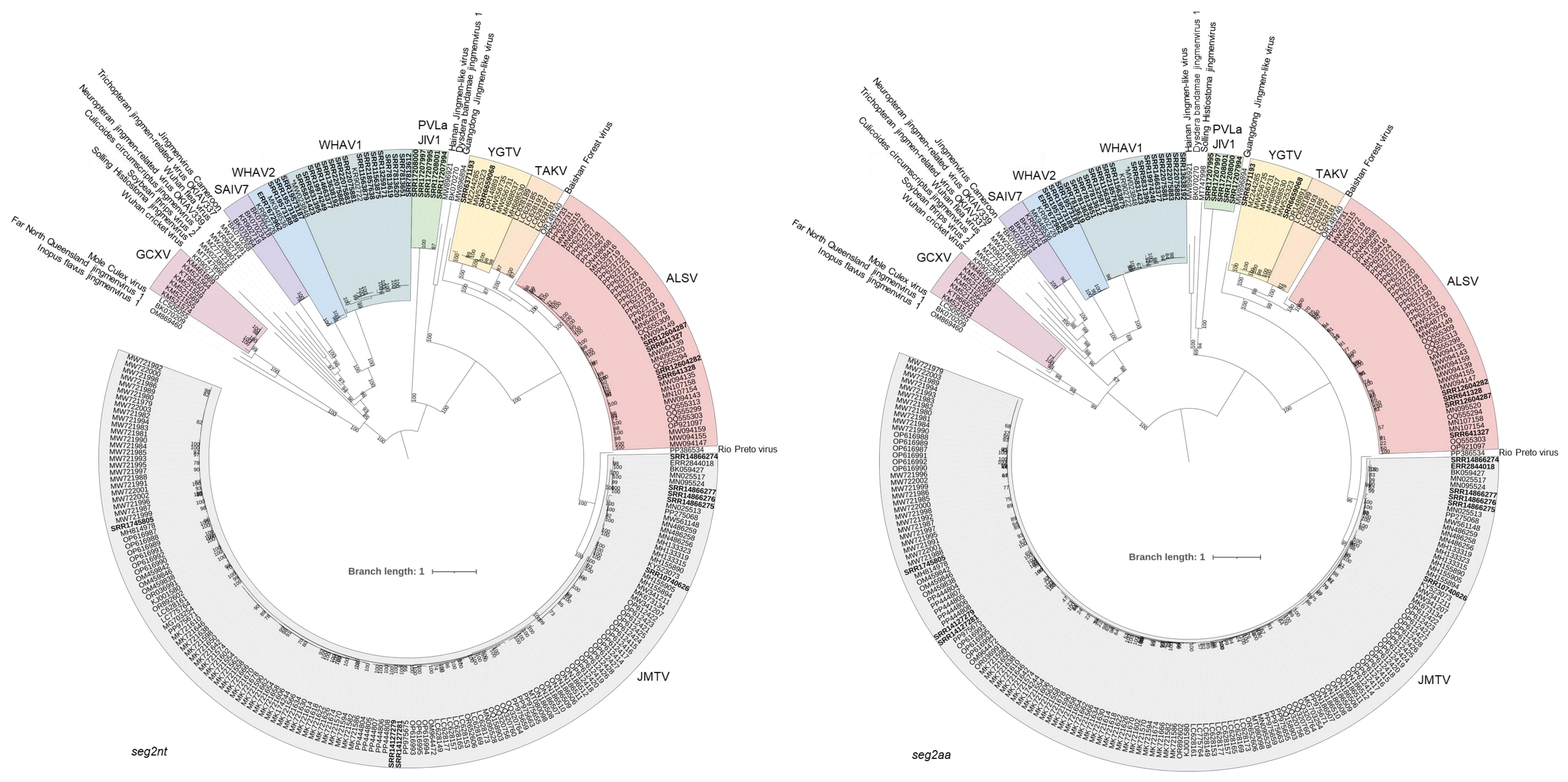

Supplementary File 2: Phylogenetic analyses of segments 1, 2 , 3 and 4 coding-complete nucleotide and amino acid sequences (whole genomes only, i.e. including at least 4 segments; n=263). The sequences were aligned with MAFFT in Geneious Prime 2024.0.7, the phylogeny was built with IQ-TREE3, using the model described in Table 5 respectively and mid-point rooted using Interactive Tree of Life. Bootstrap support values above 70 were included as branch labels. The scale bar represents substitutions per site. The sequences identified by our criteria as part of the same species are highlighted in differently coloured ranges. Sequences that are not in a coloured range represent the only complete genome of their species. The two sequences which we could not conclusively classify using our criteria are preceded by an asterisk *. The sequences assembled in this study are highlighted in bold. JMTV: Jīngmén tick virus, ALSV: Ālóngshān virus, TAKV: Takashi virus, YGTV: Yánggōu tick virus, PVLaJIV1: Plasmopara viticola lesion associated Jingman-like virus 1, WHAV1: Wǔhàn aphid virus 1, WHAV2: Wǔhàn aphid virus 2,SAIV7: Shuāngào insect virus 7, GCXV: Guaico Culex virus.

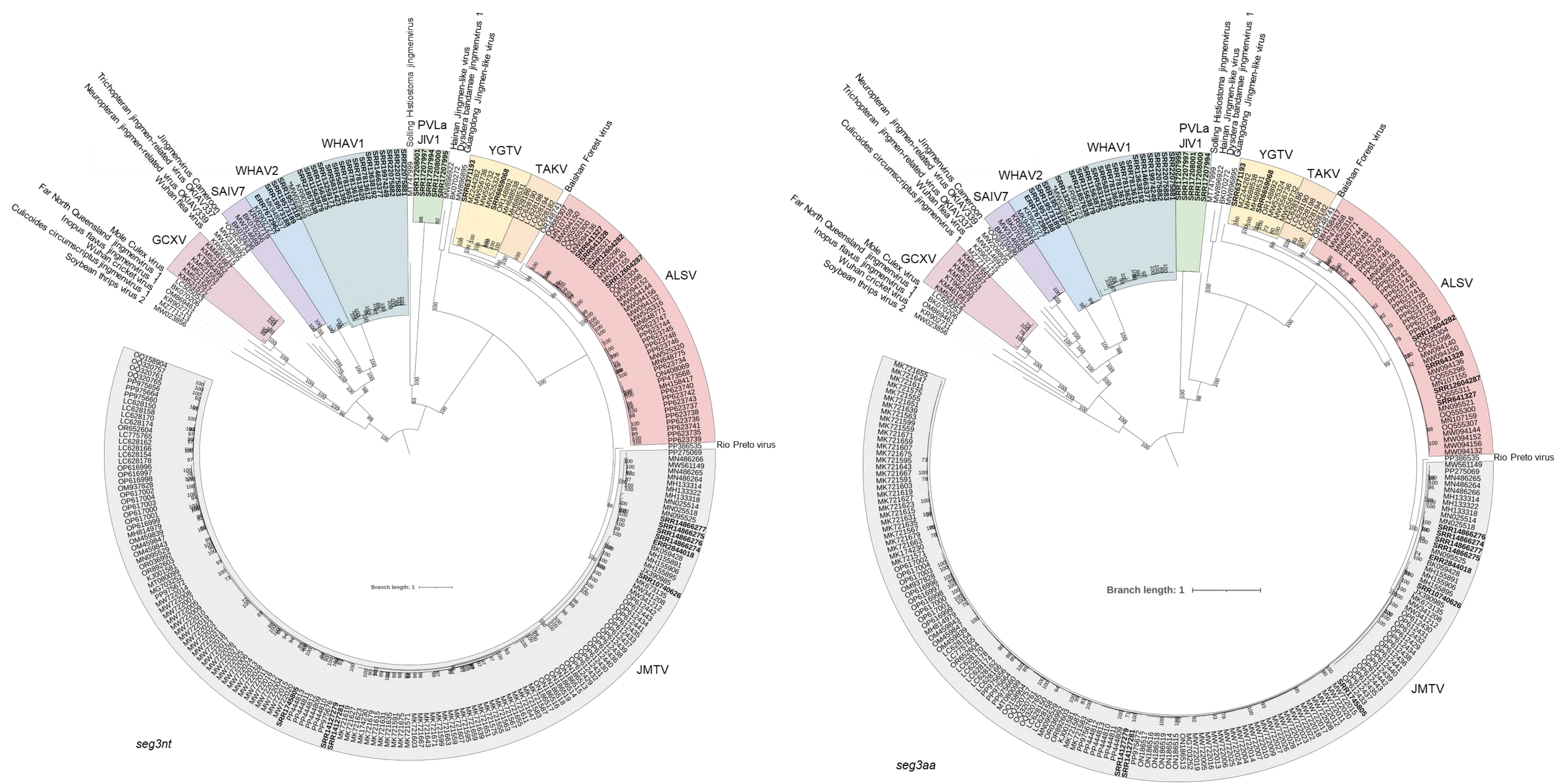

Supplementary File 2: Phylogenetic analyses of segments 1, 2 , 3 and 4 coding-complete nucleotide and amino acid sequences (whole genomes only, i.e. including at least 4 segments; n=263). The sequences were aligned with MAFFT in Geneious Prime 2024.0.7, the phylogeny was built with IQ-TREE3, using the model described in Table 5 respectively and mid-point rooted using Interactive Tree of Life. Bootstrap support values above 70 were included as branch labels. The scale bar represents substitutions per site. The sequences identified by our criteria as part of the same species are highlighted in differently coloured ranges. Sequences that are not in a coloured range represent the only complete genome of their species. The two sequences which we could not conclusively classify using our criteria are preceded by an asterisk *. The sequences assembled in this study are highlighted in bold. JMTV: Jīngmén tick virus, ALSV: Ālóngshān virus, TAKV: Takashi virus, YGTV: Yánggōu tick virus, PVLaJlV1: Plasmopara viticola lesion associated Jingman-like virus 1, WHAV1: Wǔhàn aphid virus 1, WHAV2: Wǔhàn aphid virus 2,SAIV7: Shuāngào insect virus 7, GCXV: Guaico Culex virus.

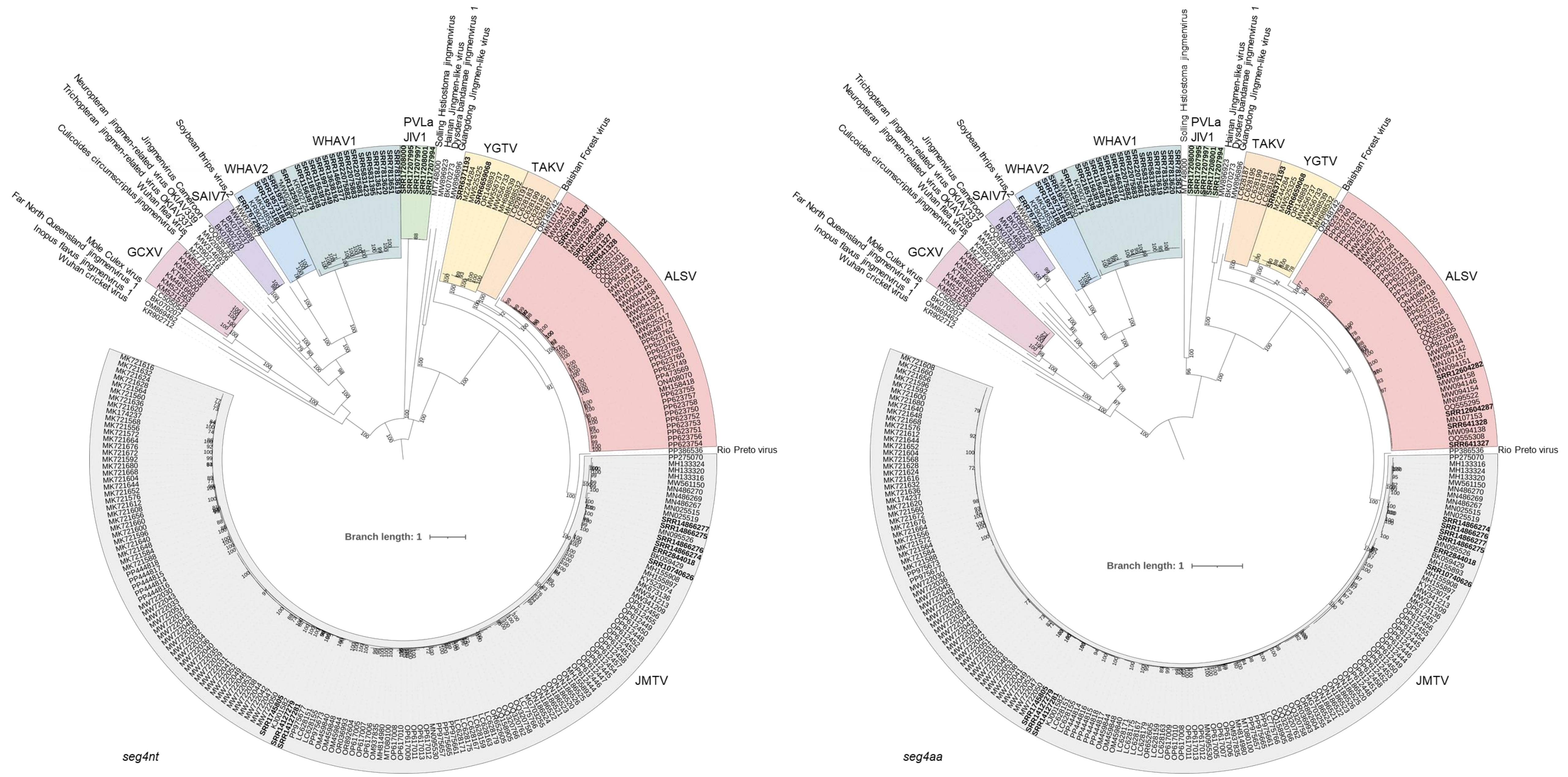

Supplementary File 2: Phylogenetic analyses of segments 1, 2 , 3 and 4 coding-complete nucleotide and amino acid sequences (whole genomes only, i.e. including at least 4 segments; n=263). The sequences were aligned with MAFFT in Geneious Prime 2024.0.7, the phylogeny was built with IQ-TREE3, using the model described in Table 5 respectively and mid-point rooted using Interactive Tree of Life. Bootstrap support values above 70 were included as branch labels. The scale bar represents substitutions per site. The sequences identified by our criteria as part of the same species are highlighted in differently coloured ranges. Sequences that are not in a coloured range represent the only complete genome of their species. The two sequences which we could not conclusively classify using our criteria are preceded by an asterisk *. The sequences assembled in this study are highlighted in bold. JMTV: Jīngmén tick virus, ALSV: Ālóngshān virus, TAKV: Takashi virus, YGTV: Yánggōu tick virus, PVLaJlV1: Plasmopara viticola lesion associated Jingman-like virus 1, WHAV1: Wǔhàn aphid virus 1, WHAV2: Wǔhàn aphid virus 2,SAIV7: Shuāngào insect virus 7, GCXV: Guaico Culex virus.
